# Mapping mental models of science communication: How academics in Germany, Austria and Switzerland understand and practice science communication

**DOI:** 10.1177/09636625211065743

**Published:** 2022-01-11

**Authors:** Sabrina Heike Kessler, Mike S. Schäfer, David Johann, Heiko Rauhut

**Affiliations:** University of Zurich, Switzerland; University of Zurich, Switzerland; ETH Zurich, Switzerland; University of Zurich, Switzerland

**Keywords:** mental models, public engagement with science, public understanding of science, science communication, strategic science communication

## Abstract

The mental models that individual scholars have of science communication – how it works, what it is supposed to achieve and so on – shape the way these academics actually communicate to the public. But these mental models, and their prevalence among scholars, have rarely been analysed. Drawing on a large-scale, representative web survey of academics at universities in Germany, Austria and Switzerland (*n* = 15,778) from 2020, we identify three mental models that are prevalent among scholars, and that correspond to conceptual models found in science communication theory: ‘Public Understanding of Science’, ‘Public Engagement with Science’ and ‘Strategic Science Communication’. The results suggest that the ‘Strategic Science Communication’ model is particularly prevalent among academics in precarious employment and female scholars. Extrinsically motivated academics, that is, those under pressure to win grants, also seem to use science communication more strategically. The ‘Public Engagement’ model is prevalent among older and female scholars, while ‘Public Understanding’ is particularly prevalent among scholars who find their work especially meaningful. Findings also reveal that academics’ mental models largely align with the way they practice science communication.

## 1. Introduction

Nobel laureates (Doherty, 2018), academics and scientific academies (ALLEA, 2019), as well as politicians and political organizations (BMBF, 2019) have all highlighted the importance of science communication in recent years – and the current COVID-19 pandemic has further increased its importance. However, science communication does not mean exactly the same to different researchers. Different ideas are circulating concerning its aims, target audiences, messages and channels, and depending on the specific concept of science communication a scholar has in mind, she or he may communicate very differently, or not at all, with the public.

These individual conceptions of science communication can be interpreted as ‘mental models’, that is, cognitive representations of an issue based on an individual’s experiences and knowledge. As Al-Diban (2012: 2020) states, ‘[m]ental models are internal representations containing meaningful declarative and procedural knowledge that people use to understand specific phenomena’. One example is the cognitive conception of how to communicate with a layperson so that they understand a complicated scientific issue. Mental models give an underlying structure to beliefs and underpin values (Gentner, 2002). Mental models represent subjective experiences, ideas, thoughts and feelings (Al-Diban, 2012; Johnson-Laird, 1983). They enable individuals to make inferences and predictions, understand phenomena, decide what action to take and control its execution (Johnson-Laird, 1983). Mental models are involved in regulating action processes and are both implicit cognitive tools for and the results of complex thinking (Al-Diban, 2012).

Representative studies of academics’ mental models about science communication are rare, especially analyses across disciplines. This study builds on conceptual developments in science communication and empirical investigations of factors that potentially explain why academics engage with non-academics. Based on large-scale, representative survey data (Rauhut et al., 2021a, 2021b), it aims to capture the mental models of scholars in Germany, Austria and Switzerland and their applied science communication across all scientific disciplines.

## 2. Three models of science communication

Science communication encompasses all communication that focuses on science, scientific work and its results (Schäfer et al., 2019). In external science communication, the non-scientific lay public is a recognized part of the audience (Davies and Horst, 2016). Communicators include scientific research institutions and public relations (PR) agencies, as well as academics themselves. This broad understanding of science communication has evolved over several decades. Based on this understanding of science communication, various conceptual models of science communication have emerged that are often grouped into three models (for an overview, see Akin, 2017; Schmid-Petri and Bürger, 2019):

- The debate started with the *Deficit Model* in the 1960s and the concept of natural science education, shifting to the paradigm of the *Public Understanding of Science* (PUS) during the 1980s and 1990s (Bucchi and Trench, 2014; Schäfer et al., 2019). Both are based on the idea of a deficit of knowledge or understanding among scientific laypersons that needs to be eliminated (Schäfer and Metag, 2021). Although the deficit model’s popularity peaked between the 1960s and 1980s (Bauer et al., 2007), and although key assumptions of the model lack empirical support (e.g. suggesting that knowledge about a scientific issue might have no effect, or even a polarizing one, on public opinion; see Akin, 2017; Schmid-Petri and Bürger, 2019), science communication is often still characterized by a deficit approach (e.g. Lee and VanDyke, 2015; Su et al., 2017). Academics who follow the PUS paradigm assume that effective science is accompanied by an increase in lay knowledge about science and scientific literacy, which is inevitably supposed to lead to greater public support for science and to legitimation for its rarefied societal position (Meyer, 2016; Schmid-Petri and Bürger, 2019). Schmidt-Petri and Bürger (2019) explain the long-lasting popularity of deficit approaches by different factors: for example, the training of academics leads them to tend to perceive their potential audiences as sharing their interests, and intuitively they apply the deficit model when interacting with a public that they assume is quick and eager to learn. Furthermore, the appeal of the deficit model stems from its simplicity and easy implementation; the problem is attributed to the public, not to science itself. Hence, the solution seems straightforward and can be addressed within the existing educational system (Schmid-Petri and Bürger, 2019; Simis et al., 2016). Finally, the model does not emphasize feedback loops and understands the science communication process as a hierarchical, top-down, one-way dissemination of knowledge (Burchell, 2015), which separates the public from scientific discourse and prevents them from influencing it (Schmid-Petri and Bürger, 2019).
- A second model emerged after 2000, aiming to initiate a two-way dialogue between science and the public: the *Public Engagement with Science* (PES) model (Bucchi and Trench, 2014; Burchell, 2015; Schäfer et al., 2019). Science and academics were no longer seen as apart from society; instead, the importance of interaction and dialogue among different stakeholders was emphasized (Akin, 2017; Schmid-Petri and Bürger, 2019). There has been a shift ‘from public awareness of science to citizen engagement, from communication to dialogue, from science and society to science in society’ (Bucchi and Trench, 2014: 4). Accordingly, there has been an increased effort to involve non-scientists in all phases of scientific research and knowledge generation (i.e. citizen science, see Meyer, 2016). Establishing a dialogue between science and society and enabling participation of non-academics became an important goal of science communication (Akin, 2017). As a consequence, programmatic and normative papers in many countries highlight the need to discuss science with the general public (Schäfer, 2009). Science communication is understood as a process among equals (Bucchi and Trench, 2014; Schäfer, 2009; Schmid-Petri and Bürger, 2019).
- Dudo and Besley (2016) as well as Besley et al. (2019) criticize the dialogue model for not specifying communication outcomes or goals, and the knowledge deficit model for focusing only on one outcome (knowledge). A major criticism of dialogic approaches in this regard is that too many science communicators use them only to fill perceived knowledge deficits. However, scholars can internalize both models about the value of increasing science literacy and about the value of dialogue. According to Nisbet and Markowitz (2016), a third model has recently gained prominence, which they have called *Strategic Science Communication* (Besley et al., 2019). In recent years, building the legitimation of science and its protagonists has become a goal of science communication. Particularly for scientific institutions, but also for individual academics, the need to legitimize themselves has become more important (e.g. Weingart, 2005). It has been argued that the increased autonomy of institutions of higher education, combined with the increasing demand for resources, necessitates greater public communication by higher-education institutions (HEIs) and individual academics (e.g. Marcinkowski et al., 2014), and that this communication tends to be strategic, favouring instrumental goals such as reputation- or image-building over the dissemination of scientific knowledge (e.g. Bauer and Gregory, 2007). Similar diagnoses are made for individual academics: In many countries, they are described as being under professional pressure to compete for the few permanent positions available, to publish extensively, to raise third-party funds, and also to use public communication to their advantage (e.g. Fang and Casadevall, 2015; Metz-Göckel et al., 2016).

The three models described above are linked to different goals, target groups, preferred messages and channels of science communication. Their importance in science politics is well established (Akin, 2017; Schmid-Petri and Bürger, 2019) and their prevalence among academic and scientific organizations and their leadership has been repeatedly analysed (e.g. Davies, 2020; Friedrichsmeier and Marcinkowski, 2016). However, it is still largely unclear to what extent individual academics have internalized these models. While qualitative analyses have described the broad spectrum of understandings of science communication among bioscientists (Rödder, 2009), political scientists (Herrmann-Giovanelli, 2013) or social scientists in general (Fähnrich and Lüthje, 2017), it is difficult to assess how widespread these models are quantitatively based on previous studies. For Spain, Llorente et al. (2019) used survey data to show that many scientists believe that the lack of public scientific knowledge is the main barrier to engaging society in public engagement activities. Llorente et al. illustrate that such a belief fits more aptly with a one-way communication approach than with approaches that focus on mutual learning processes or public engagement activities. Other quantitative studies have suggested a general perseverance of deficit model approaches among communicators engaged in science communication (Ridgway et al., 2020) and among scholars in particular, but these studies were limited to single universities (Simis et al., 2016), government agencies (Lee and VanDyke, 2015) or science organizations (Su et al., 2017). It is therefore important to investigate the prevalence of the three outlined models among academics using a comprehensive, cross-national and interdisciplinary sample – which is what the present study does.

## 3. Factors explaining how scholars do science communication

In addition to mapping scholars’ mental models of science communication, we will analyse the practices of science communication with which these models are correlated. Many previous studies have analysed the factors that lead academics to interact with the public. Such studies exist for countries like Argentina (Kreimer et al., 2011), France (Jensen, 2011), Germany (Ivanova et al., 2013; Marcinkowski et al., 2014; Peters, 2009), Norway (Kyvik, 2005), Switzerland (Crettaz von Roten, 2011), the United Kingdom (Poliakoff and Webb, 2007) and the United States (Besley, 2015; Dudo and Besley, 2016; Dunwoody et al., 2009), among others, as well as across countries (Bentley and Kyvik, 2011; Entradas and Bauer, 2019; Peters et al., 2008). Some have analysed academics from various disciplines (e.g. Crettaz von Roten, 2011; Kreimer et al., 2011; Kyvik, 2005; Poliakoff and Webb, 2007), while others focus rather on natural scientists (Besley, 2015; Dudo and Besley, 2016) or specific disciplines like astronomy (Entradas and Bauer, 2019), bioscience (Dunwoody et al., 2009; Peters et al., 2008; Rödder, 2009) and climate science (Ivanova et al., 2013; Post, 2016).

Based on different theoretical approaches, such as the Orientation1–Stimulus–Orientation2–Response model (Dudo, 2013) or the theory of planned behaviour (e.g. Besley et al., 2013), previous studies suggest that different factors influence scholars’ communication activities. On the level of individual researchers, studies have shown that researchers’ attitudes towards public communication are important (for an overview, see Besley et al., 2018). It has been demonstrated, for example, that researchers with positive attitudes towards public engagement are more likely to undertake communication activities (Dudo, 2013; Poliakoff and Webb, 2007). Furthermore, intrinsic factors, such as the *communicative self-efficacy* of academics and the feeling of contributing to society or fulfilling a sense of responsibility, are also related to the intensity of external contacts (Allgaier et al., 2013; Besley et al., 2013, 2018; Dudo, 2013). Findings related to the perceived social norms of academics – usually operationalized as perceptions of peer expectations – are mixed (Besley, 2015; Besley et al., 2013; Gascoigne and Metcalfe, 1997; Poliakoff and Webb, 2007). External factors also play a role. The absence of career incentives (Jacobson et al., 2004) or funding (Allgaier et al., 2013), in addition to a lack of time (Allgaier et al., 2013), negatively impacts the motivation to engage in external science communication.

Findings on sociodemographic factors, such as age and gender, are mixed. The participation of women in public engagements may be slightly higher than that of men (Burchell, 2015). Nationality seems to be a predictor (Bentley and Kyvik, 2011; Entradas and Bauer, 2019; Neresini and Bucchi, 2011; Peters, 2009; Peters et al., 2008; Rauchfleisch et al., 2021). Evidence suggests that older researchers communicate more with news media (Burchell, 2015), while social media engagement is more common among younger academics (Pew Research Center, 2015). Furthermore, the status of the individual researchers – measured, for example, by the number of publications (Bentley and Kyvik, 2011; Entradas and Bauer, 2019) or management positions (Entradas and Bauer, 2019; Ivanova et al., 2013) – is a key factor (see Besley et al., 2018). Previous studies suggest a positive correlation between the academics’ status and the number of their media interactions (Bauer and Jensen, 2011; Dudo, 2013; Dunwoody et al., 2009).

The scientific fields in which academics work also seem to play a role (Besley et al., 2013, 2018; Kreimer et al., 2011; Yeo and Brossard, 2017). Burchell (2015) reports notable differences between science, technology, engineering and mathematics (STEM), on one hand, and other disciplines, on the other. This appears to shape contemporary understandings of academics and participation in public engagement among them: participation is considerably higher in the arts, humanities and social sciences than in STEM subjects (Burchell, 2015).

In contrast to individual and disciplinary characteristics, organizational influences have rarely been considered in research (for an overview, see Entradas and Bauer, 2019; Marcinkowski et al., 2014).

## 4. Research questions

The three models of science communication outlined above are associated with different goals, definitions of quality and, potentially, practices of science communication that academics may have internalized.

First, we analyse these mental models among academics in Germany, Austria and Switzerland, before asking as follows:

*RQ1.* Among which academics in Germany, Austria and Switzerland do the models ‘Public Understanding of Science’, ‘Public Engagement with Science’ and ‘Strategic Science Communication’ predominate?

Since mental models and attitudes towards science communication can be related to behaviour (Besley et al., 2018), we also ask:

*RQ2*. What types of science communication behaviour are these mental models associated with?

## 5. Method

### Data

Eliciting mental models is often done by the direct method of interviews or questionnaires that explicitly ask people about their beliefs (Gentner, 2002). Using a similar approach, we conducted a large-scale and representative web survey among academics (Rauhut et al., 2021a, 2021b), that is, among researchers and lecturers from all academic disciplines (including humanities) working at HEIs in Germany, Austria and Switzerland (DACH region); these represent our statistical population (universe).^1^ Before collecting the contact data, we compiled an overview of the number of scientists working at the various HEIs in the three countries. Student assistants researched the email addresses via the HEI websites, with each student assistant researching between 10,000 and 15,000 contact details. A full survey of addresses was sought for all three countries. The collection of addresses for Germany was done in cooperation with the DZHW. Of the addresses researched for Germany, a 50% sample was drawn up for our study (the other half of the addresses were used by the DZHW for their own study). In total, we had 140,953 valid addresses of scientists who were invited by email to participate in the survey. If the target persons did not participate after the first invitation email, up to two reminder emails were sent out (Rauhut et al., 2021b).

The field time was from 14 February 2020 to 30 April 2020. The survey data comprise 15,778 academics from 236 institutions (response rate: about 11%); 8182 respondents came from Germany, 2771 from Austria and 4825 from Switzerland. Due to the sensitive nature of our data, we applied for, and received, the approval of the university’s ethics commission in September 2019 (Rauhut et al., 2021b). Since no academics at universities of applied sciences were surveyed in Germany, those who stated that they worked at universities of applied sciences were excluded from the analysis for reasons of better comparability between the three countries (Johann et al., 2021). This reduces the number of cases by *N* = 914.

### Sample description

Table 1 displays the summary statistics: 40% of the respondents were between 30 and 39 years old. More men (56%) than women participated in the survey. Most respondents had a fixed-term contract (69%) and were employed full-time (63%)^2^: 42% of the respondents were predoctoral researchers, 38% were postdoctoral researchers, and 21% were professors. While 26% of respondents indicated the social sciences as their field of research, 18% natural sciences, 17% life sciences, 14% engineering, another 16% of respondents indicated the humanities as their field of research. The respondents reported spending more than 1.5 hours a week on science communication, but stated they would like to spend almost twice as much time on it. As for the perceived work situation, 80%–90% agreed somewhat or completely with the statements, ‘My work is meaningful’ and ‘I enjoy considerable autonomy in my everyday working life’. Around 60% agreed somewhat or completely with the statements ‘The competition among those working in my discipline is intense’ and ‘My workload is excessive’.

**Table 1.** Question wording, coding and summary statistics.

Variables	Characteristics/values	Mean and standard deviation
Age	Younger than 30, between 30 and 39, between 40 and 49, between 50 and 59, 60 or older	Younger than 30: *M* *=* 0.21 (21%), *SD* = 0.41 Between 30 and 39: *M* *=* 0.40 (40%), *SD* = 0.49 Between 40 and 49: *M* *=* 0.17 (17%), *SD* = 0.37 Between 50 and 59: *M* *=* 0.14 (14%), *SD* = 0.35 60 or older: *M* *=* 0.07 (7%), *SD* = 0.26
Gender	0 = male; 1 = female	*M* *=* 0.44, *SD* = 0.50
Country of residence	Germany, Austria, Switzerland	Germany: *M* *=* 0.55, *SD* = 0.50 Austria: *M* *=* 0.17, *SD* = 0.38 Switzerland: *M* *=* 0.28, *SD* = 0.45
Contract	0 = not tenured, 1 = tenured	*M* *=* 0.31, *SD* = 0.46
Part-time employment	0 = full time, 1 = part time	*M* *=* 0.37, *SD* = 0.48
Academic status	Predoctoral researcher, postdoctoral researcher, professor	Predoc: *M* *=* 0.42, *SD* = 0.49 Postdoc: *M* *=* 0.38, *SD* = 0.48 Professor: *M* *=* 0.21, *SD* = 0.40
Perceived competition	‘The competition among those working in my discipline is intense’. Scale ranging from 1 (don’t agree at all) to 6 (agree completely)	*M* *=* 3.85, *SD* = 1.52
Perception of work as meaningful	‘My work is meaningful’. Scale ranging from 1 (don’t agree at all) to 6 (agree completely)	*M* *=* 4.58, *SD* = 1.27
Perceived workload	‘My workload is excessive’. Scale ranging from 1 (don’t agree at all) to 6 (agree completely)	*M* *=* 3.95, *SD* = 1.43
Perceived autonomy in everyday work	‘I enjoy considerable autonomy in my everyday working life’. Scale ranging from 1 (don’t agree at all) to 6 (agree completely)	*M* *=* *5.00, SD* = 1.10
Perceived pressure to publish	‘In my subject area, there is considerable pressure to publish’. Scale ranging from 1 (don’t agree at all) to 6 (agree completely)	*M* *=* 4.63, *SD* = 1.32
Perceived pressure to win grants	‘In my subject area, there is considerable pressure to attract third-party funding’. Scale ranging from 1 (don’t agree at all) to 6 (agree completely)	*M* *=* 4.30, *SD* = 1.54
Science field in which one is active	Humanities Social sciences Natural sciences Life sciences Engineering Other field	Humanities: *M* *=* 0.16, *SD* = 0.37 Social sciences: *M* *=* 0.26, *SD* = 0.44 Natural science: *M* *=* 0.18, *SD* = 0.39 Life sciences: *M* *=* 0.17, *SD* = 0.38 Engineering: *M* *=* 0.14, *SD* = 0.35 Other field: *M* *=* 0.08, *SD* = 0.28
Discrepancy between desired time for research and time actually available for research^a^	The variable can take values between −1 and 1. Values below 0 indicate that respondents desire less time for research than they actually have at their disposal; values above 0 indicate that researchers desire more time for research than is actually available to them	*M* *=* 0.14, *SD* = 0.24
Time spent on science communication	Current and desired time expenditure in hours per week	Current: *M* *=* 1.75, *SD* = 3.33 Desired: *M* *=* 3.11, *SD* = 4.34

a This variable is taken from Johann et al. (2021).

With regard to deviations of the sample from the statistical population (researchers and lecturers from all academic disciplines working at HEI in the DACH region), it can first be said that professors are overrepresented in the sample of all three countries, while junior and senior academics are underrepresented. Moreover, in the Swiss data, female academics are slightly overrepresented. Furthermore, there are some deviations with regard to discipline: At least in Germany and Switzerland, researchers from the humanities, social sciences and natural sciences are overrepresented, while researchers from medicine are underrepresented (Rauhut et al., 2021b; see also the data provided by the German Federal Statistical Office (Statistisches Bundesamt, 2019), the Austrian Federal Statistical Institute (Bundesanstalt Statistik Österreich, 2019) and the Swiss Federal Statistical Office (Bundesamt für Statistik, 2019)). We take these differences into account in our multivariate analyses by controlling for status, gender and scientific discipline (Rauhut et al., 2021b).^3^

### Survey instrument and main measurements

The survey began with questions about the participants’ sociodemographics, such as gender, country of residence and age. The next part concerned academic work and research situations. These questions aimed at classifying the academics by their academic position, status, career and discipline (Rauhut et al., 2021b). We were interested in how the respondents perceived several aspects of their working conditions and workload and the meaningfulness of their work.

To gain insights into how academics think about and perform science communication, we integrated two item batteries that ask about (a) their perceptions and (b) their practice of science communication:

- To measure the subjective perceptions of science communication, 13 items were designed in relation to the three basic models, PUS, PES and Strategic Science Communication (see Table 2). Items relating to PUS (e.g. ‘If members of the public understand my research, they judge it positively’, ‘If members of the public are hostile towards my research, I can change their minds with facts’ and ‘My main task in public communication is to educate the public’) were constructed with reference to Simis et al. (2016) and Peters (2009). Items relating to Strategic Science Communication (e.g. ‘I find it important to develop a communication strategy, because this makes it easier for me to attract funding’ and ‘I think that communication with the public has a positive impact on my academic career’) were constructed with reference to Besley et al. (2019) and Nisbet and Markowitz (2016). Items relating to PES (e.g. ‘Direct dialogue with the public about my research is important to me’ and ‘Dialogue with the public is instructive for me, too’) were constructed with reference to Bucchi and Trench (2014), Peterman et al. (2017), Schäfer (2009) and Schmid-Petri and Bürger (2019). The respondents were asked to indicate how much they agreed with each of the 13 items on a scale from 1 to 6. In order to confirm the theoretically founded relationships, an exploratory factor analysis was conducted which included all items on the subjective conceptions of science communication (see Table 2). Both the Bartlett test (χ²(78) = 27,578.33, *p* < .001) and the Kaiser–Meyer–Olkin measure of sampling adequacy (KMO = .897) indicate that the variables are suitable for factor analysis. We performed a principal-component factor analysis with varimax rotation, which indicated the presence of three factors with eigenvalues greater than 1.0. We opted for the three-factor solution based on this finding, a scree plot, as well as theoretical considerations (e.g. Bartholomew et al., 2011). The three-factor solution explains 52.68% of the variance. The three factors map the theoretical expectations about the three mental models Strategic Science Communication, PES and PUS. For the multivariate analyses, we calculated and used the factor scores (see Table 2).
- To enquire about the academics’ *actual* science communication (what we call the practice of science communication), we determined how and through which channels they conduct science communication, that is, what experience they themselves have with it (see Table 3). To measure the practice of science communication, we use six items: (a) ‘Conversations with members of the public give me inspiration for my research’, (b) ‘I have had controversial discussions with members of the public about my research’, (c) ‘I use social media such as YouTube, Twitter or Facebook to inform the public about my research’, (d) ‘I discuss my research with other users on social media such as YouTube, Twitter or Facebook’, (e) ‘I prefer to explain details of my research to the public than to discuss what it means for society’ and (f) ‘When I communicate with members of the public, I try to present my area of research as positively as possible’. We performed a principal-component factor analysis with varimax rotation to test which latent dimensions are represented by the six items. The six items represent three latent dimensions that can be described as (a) PES communication behaviour, (b) strategic communication behaviour and (c) PUS communication behaviour (see Table 3). The three factors explain 72.16% of the variance. For the multivariate analyses, we calculated and used the factor scores (see Table 3).

**Table 2.** Items measuring mental models of science communication and factor analysis.

Question wording: The following section is about how you see your role as an academic in society. How much do you agree with the following statements?	Mean (standard deviation)	Rotated factor loadings^a^			Dimension	Min/max of factor scores
		Component				
		1	2	3		
I find it important to develop a communication strategy, because this makes it easier for me to attract funding.^b^	3.5 (1.5)	0.77	−0.17	0.07	Strategic science communication	Min: −4.14 Max: 2.91
Even when planning a project, I think about how I can communicate my research findings to the public.^b^	3.0 (1.6)	0.70	0.23	0.12		
I think that communication with the public has a positive impact on my academic career.^b^	3.7 (1.5)	0.63	0.15	0.26		
It’s important to me to communicate my research findings to the public.^b^	4.2 (1.4)	0.53	0.47	0.31		
I find it more important to concentrate on research and teaching than to communicate with the public.^c^	3.4 (1.5)	0.21	0.72	0.06	Public engagement with science	Min: −3.13 Max: 3.15
Scientific findings and models should only be discussed within the scientific community.^c^	5.0 (1.2)	−0.17	0.66	0.09		
Science communication should be carried out by journalists or press offices, not by me.^c^	4.3 (1.4)	0.09	0.62	−0.08		
Direct dialogue with the public about my research is important to me.^b^	3.7 (1.5)	0.45	0.57	0.39		
Dialogue with the public is instructive for me, too.^b^	4.4 (1.4)	0.34	0.50	0.41		
If members of the public understand my research, they judge it positively.^b^	4.6 (1.2)	0.10	0.02	0.76	Public understanding of science	Min: −3.77 Max: 3.19
If members of the public are hostile towards my research, I can change their minds with facts.^b^	3.7 (1.3)	0.09	0.07	0.73		
My main task in public communication is to educate the public.^b^	3.3 (1.5)	0.25	0.24	0.47		
When I communicate with members of the public, I try to get them actively involved.^b^	3.9 (1.4)	0.34	0.39	0.45		

a Extraction method: principal-component factor method; Rotation: orthogonal varimax (after Kaiser normalization).

b Values 1 (do not agree at all) to 6 (agree completely).

c The items were recoded to 1 (agree completely) to 6 (do not agree at all). Reported means and factor loadings are based on the recoded items.

**Table 3.** Items measuring respondents’ practice of science communication and factor analysis.

Question wording: The following section is about your own experiences with science communication. How much do you agree with the following statements?	*M* (*SD*)^a^	Rotated factor loadings^b^			Dimension	Min/max of factor scores
		Component				
		1	2	3		
I use social media such as YouTube, Twitter or Facebook to inform the public about my research.	1.92 (1.47)	0.92	0.14	0.06	Strategic communication	Min: −1.04 Max: 3.44
I discuss my research with other users on social media such as YouTube, Twitter or Facebook.	1.76 (1.32)	0.93	0.10	0.04		
Conversations with members of the public give me inspiration for my research.	3.36 (1.61)	0.16	0.79	0.06	PES communication	Min: −2.48 Max: 2.45
I have had controversial discussions with members of the public about my research.	3.06 (1.73)	0.07	0.81	0.00		
I prefer to explain details of my research to the public than to discuss what it means for society.	2.70 (1.41)	0.08	−0.17	0.81	PUS communication	Min: −2.62 Max: 2.56
When I communicate with members of the public, I try to present my area of research as positively as possible.	4.25 (1.31)	0.01	0.27	0.72		

PES: *Public Engagement with Science*; PUS: *Public Understanding of Science*.

a Values 1 (do not agree at all) to 6 (agree completely).

b Extraction method: principal-component factor method; Rotation: orthogonal varimax (after Kaiser normalization).

## 6. Results

### Mental models of science communication (RQ1)

Respondents widely agreed on several items about science communication: 70% agreed (including those who ‘somewhat agreed’) with the statement, ‘It is important to me to communicate my research findings to the public’. More than 80% agreed with the statement, ‘If members of the public understand my research, they judge it positively’ (84%). More than 60% agreed with the statement, ‘When I communicate with members of the public, I try to get them actively involved’ (64%), and more than 75% agreed with the statement ‘Dialogue with the public is instructive for me too’ (77%). The last two items indicate a general orientation towards and internalization of the PES model. However, a strategic idea also appears, as the importance of dialogical communication for the academics themselves is revealed several times. In addition, a conception of PUS becomes clear; the majority of the academics assumes that citizens would evaluate their research more positively if they understood it. The statements that were most strongly rejected were, ‘Scientific findings and models should only be discussed within the scientific community’ and ‘Science communication should be carried out by journalists or press offices, not by me’. Thus, the majority of academics see themselves primarily in the role of active science communicators for the public.

Having internalized one model does not exclude having internalized one of the other models. However, the means (and standard deviations) of the indicators used to measure the different mental models indicate that the mental models were not internalized equally across academics. The indicators of the PES model obtain the highest mean values, which suggests that the PES model is more prevalent among academics than the other mental models.^4^

We calculated multivariate regression analyses to capture how much variance of the models could be explained by sociodemographic factors, academic status, employment conditions, perceived work situation and scientific field. The multivariate regression analyses indicate that 8% of the variance of the Strategic Science Communication model, 6% of the variance of the PES model and 4% of the variance of the PUS model can be explained by sociodemographic factors, academic status, employment conditions, perceived work situation and scientific field (Table 4; and see Supplemental Material, Tables A1 to A3).^5^

**Table 4.** Factors explaining mental models of science communication (OLS regression models; unstandardized coefficients).

	Strategic Science Communication (factor scores)	PES (factor scores)	PUS (factor scores)
Female (Ref.: Men)	.12*** (0.02)	.14*** (0.02)	−.00 (0.02)
30–39 (Ref.: <30)	.04 (0.04)	.07 (0.04)	−.00 (0.04)
40–49 (Ref.: <30)	.02 (0.05)	.19*** (0.05)	−.02 (0.05)
50–59 (Ref.: <30)	−.02 (0.05)	.33*** (0.05)	.00 (0.05)
60+ (Ref.: <30)	.02 (0.06)	.39*** (0.06)	−0.00 (0.06)
Austria (Ref.: Germany)	.19*** (0.03)	−.00 (0.03)	.04 (0.03)
Switzerland (Ref.: Germany)	.25*** (0.03)	0.04 (0.03)	.01 (0.03)
Postdoc (Ref.: Predoc)	−.11*** (0.03)	−.05 (0.03)	−.06 (0.03)
Professor (Ref.: Predoc)	−.13** (0.05)	−.07 (0.05)	−.04 (0.05)
Tenured (Ref.: Not tenured)	−.11** (0.03)	.03 (0.04)	.06 (0.04)
Part-time (Ref.: Full-time)	.02 (0.03)	.04 (0.03)	.05 (0.03)
Discrepancy between desired time for research and time actually available for research	−.41*** (0.05)	−.44*** (0.05)	−.15** (0.05)
Autonomy	−.04*** (0.01)	−.00 (0.01)	.02 (0.01)
Sense foundation	.12*** (0.01)	.03** (0.01)	.14*** (0.01)
Competition	.06*** (0.01)	−.02* (0.01)	.00 (0.01)
Workload	.01 (0.01)	.02** (0.01)	.03*** (0.01)
Pressure to publish	−.03* (0.01)	−.01 (0.01)	.03* (0.01)
Pressure to win grants	.08*** (0.01)	−.02 (0.01)	.00 (0.01)
Humanities (Ref.: Natural sciences)	.21*** (0.04)	.32*** (0.04)	−.13*** (0.04)
Social sciences (Ref.: Natural sciences)	.14*** (0.04)	.37*** (0.04)	−.06 (0.04)
Life sciences (Ref.: Natural sciences)	.13*** (0.04)	.09* (0.04)	.12*** (0.04)
Engineering (Ref.: Natural sciences)	.24*** (0.04)	−.01 (0.04)	−.01 (0.04)
Other (Ref.: Natural sciences)	.31*** (0.05)	.30*** (0.05)	.05 (0.05)
Constant	−1.01*** (0.08)	−.36*** (0.08)	−1.03*** (0.09)
*N*	7592	7592	7592
Adj. *R*^2^	.08	.06	.04

PES: *Public Engagement with Science*; PUS: *Public Understanding of Science*.

Standard errors in parentheses.

* *p* < .05, **p < .01, ***p < .001.

Looking at the regression coefficients, the results indicate that it is more likely for female academics and academics from Austria and Switzerland to have internalized the Strategic Science Communication model than male academics and academics from Germany. In terms of professional status and employment conditions, predoctoral researchers and researchers who are not yet permanent employees are more likely to adhere to this model. With regard to the work situation, the results suggest that those who perceive intense competition and high pressure to obtain external funding are more likely to have internalized the Strategic Science Communication model. Moreover, the greater the discrepancy between the desired time for research and the time actually available for research (due to teaching commitments and administration), the less they have internalized the need to communicate strategically. Finally, humanities, social sciences, life sciences, engineering scholars and scientists from other fields are more inclined to the Strategic Science Communication model than natural scientists.

In terms of sociodemographic factors, female academics and older academics in particular have internalized the PES model. With regard to academic status, no statistically significant differences were found. Regarding the perceived work situation, the PES model is internalized primarily by those who see meaning in their work and less internalized among those who have too little time for research due to teaching commitments and administration. Furthermore, researchers in the life science, humanities, social sciences and other research fields are more inclined than natural scientists to adhere to this mental model.

Researchers who perceive their work as meaningful or who have a high perceived workload are more likely to have internalized the PUS model than their counterparts. Moreover, the greater the discrepancy between the desired time for research and the time actually available for research, the less they have internalized the PUS Model. Finally, researchers in the life sciences are more inclined than natural scientists to adhere to this mental model, while researchers in the humanities are less inclined than natural scientists to adhere to this mental model.

### Relationship between mental models of science communication and respondents’ practice of science communication (RQ2)

With regard to actual science communication, the indicators used indicate that strategic science communication practices are the least prominent, with PES and PUS communication practices being more common (see Table 3).

The multivariate analyses further reveal that older scientists in particular use PES communication practices, while social media (i.e. strategic communication practices) are more likely to be used by young academics for science communication. Compared with predocs, postdocs and professors tend to communicate strategically, but they use PES communication strategies to a lesser extent. The results also indicate that strategic science communication practices are more widespread in Austria and Switzerland than in Germany.

The main findings are that (a) mental models can explain a significant portion of the variance in actual communication behaviour and (b) the academics’ mental models have a significant effect on actual science communication behaviour. Those who have internalized Strategic Science Communication actually communicate particularly strategically; those who have internalized the PUS model particularly use means of a PUS communication strategy, while they tend to stay away from a PES communication strategy; and those who have internalized the PES model use all three communication strategies, but in particular means of the PES communication strategy (see Table 5).

**Table 5.** How mental models of science communication affect actual communication behaviour (OLS regression models; unstandardized coefficients).

	Strategic communication		PES communication		PUS communication	
	Basic model	Full model	Basic model	Full model	Basic model	Full model
Female (Ref.: Men)	−.08** (0.03)	−.13*** (0.03)	.05* (0.02)	−.04* (0.02)	.01 (0.02)	.01 (0.02)
30–39 (Ref.: <30)	−.04 (0.04)	−.06 (0.04)	.10** (0.04)	.05 (0.03)	−.06 (0.04)	−.06 (0.04)
40–49 (Ref.: <30)	−.12* (0.05)	−.15** (0.05)	.17*** (0.05)	.09* (0.04)	−.06 (0.05)	−.04 (0.05)
50–59 (Ref.: <30)	−.36*** (0.06)	−.40*** (0.06)	.32*** (0.05)	.20*** (0.05)	−.07 (0.05)	−.03 (0.05)
60+ (Ref.: <30)	−.54*** (0.07)	−.59*** (0.07)	.39*** (0.06)	.23*** (0.05)	.02 (0.06)	.06 (0.06)
Austria (Ref.: Germany)	.16*** (0.03)	.11*** (0.03)	.06* (0.03)	−.00 (0.03)	.03 (0.03)	.01 (0.03)
Switzerland (Ref.: Germany)	.18*** (0.03)	.12*** (0.03)	.04 (0.03)	−.04 (0.02)	.07** (0.03)	.05 (0.03)
Postdoc (Ref.: Predoc)	.12*** (0.04)	.16*** (0.04)	−.12*** (0.03)	−.06* (0.03)	−.02 (0.03)	−.00 (0.03)
Professor (Ref.: Predoc)	.21*** (0.05)	.24*** (0.05)	−.16*** (0.05)	−.09* (0.04)	−.03 (0.05)	−.02 (0.05)
Tenured (Ref.: Not tenured)	−.11** (0.04)	−.09* (0.04)	−.01 (0.04)	.00 (0.03)	.00 (0.04)	.01 (0.04)
Part-time (Ref.: Full-time)	.01 (0.03)	−.00 (0.03)	.07* (0.03)	.04 (0.02)	−.02 (0.03)	−.02 (0.03)
Discrepancy between desired time for research and time actually available for research	−.12* (0.06)	.03 (0.06)	−.13* (0.05)	.16*** (0.04)	−.01 (0.05)	.01 (0.05)
Autonomy	−.02 (0.01)	−.01 (0.01)	−.01 (0.01)	−.01 (0.01)	.03** (0.01)	.03** (0.01)
Sense foundation	.07*** (0.01)	.03** (0.01)	.12*** (0.01)	.04*** (0.01)	.04*** (0.01)	.00 (0.01)
Competition	.06*** (0.01)	.05*** (0.01)	−.01 (0.01)	−.02** (0.01)	.02* (0.01)	.01 (0.01)
Workload	.03*** (0.01)	.03** (0.01)	.04*** (0.01)	.02** (0.01)	.01 (0.01)	.00 (0.01)
Pressure to publish	−.02 (0.01)	−.01 (0.01)	.01 (0.01)	.01 (0.01)	.01 (0.01)	.01 (0.01)
Pressure to win grants	.01 (0.01)	−0.00 (0.01)	0.03** (0.01)	0.01 (0.01)	0.02** (0.01)	0.01 (0.01)
Humanities (Ref.: Natural sciences)	0.17*** (0.04)	.10* (0.04)	.47*** (0.04)	.34*** (0.03)	−.06 (0.04)	−.03 (0.04)
Social sciences (Ref.: Natural sciences)	.18*** (0.04)	.11** (0.04)	.60*** (0.04)	.46*** (0.03)	−.37*** (0.04)	−.33*** (0.04)
Life sciences (Ref.: Natural sciences)	.06 (0.04)	.01 (0.04)	.20*** (0.04)	.10** (0.03)	−.05 (0.04)	−.07 (0.04)
Engineering (Ref.: Natural sciences)	.08 (0.05)	.02 (0.04)	.13** (0.04)	.08* (0.04)	−.07 (0.04)	−.09* (0.04)
Other (Ref.: Natural sciences)	.26*** (0.05)	.15** (0.05)	.45*** (0.05)	.26*** (0.04)	−.09 (0.05)	−.10* (0.05)
Mental model Strategic Science Communication (factor scores)		.23*** (0.01)		.26*** (0.01)		.12*** (0.01)
Mental model PES (factor scores)		.11*** (0.01)		.36*** (0.01)		−.11*** (0.01)
Mental model PUS (factor scores)		.06*** (0.01)		.26*** (0.01)		.16*** (0.01)
Constant	−.61*** (0.09)	−.26** (0.09)	−1.13*** (0.09)	−.44*** (0.07)	−.40*** (0.09)	−.15 (0.09)
*N*	6947	6947	6947	6947	6947	6947
Adj. *R*^2^	.05	.10	.11	.35	.03	.08

PES: *Public Engagement with Science*; PUS: *Public Understanding of Science*.

Standard errors in parentheses.

* *p* < .05, **p < .01, ***p < .001.

To understand the *strategic* communication behaviour of academics better, we additionally divided academics into three types of researchers with different types of communication behaviour, before then estimating a multinomial logistic regression model, with the types of researchers as the dependent variable (see Supplemental Material, Table A4 and Figure A1). The dependent variable distinguishes between (a) those who do not communicate strategically (66.3%) but may or may not use other forms of communication; (b) those who communicate exclusively strategically (7.5%); and (c) those who communicate strategically, while also using at least one other form of communication (PUS and/or PES) (26.2%). The bases for the classification were the factor scores: factor scores less than or equal to 0 were assessed as ‘respondents tend *not* to use the communication strategy’, and factor scores greater than 0 were assessed as ‘respondents tend to use the communication strategy’. Academics who do not communicate strategically are the reference category.

Not only does it appear that those who have internalized the mental model of Strategic Science Communication do indeed communicate more strategically, but also that having internalized the PUS or PES mental models prevents academics from communicating *exclusively* strategically (see Figure A1 in the Supplemental Material).

## 7. Discussion

Different models of science communication are associated with different goals, definitions of quality and practices of science communication. Our results suggest that three different mental models of science communication are prevalent among academics in the DACH region: in addition to the traditional PUS and PES models, the Strategic Science Communication model is also prevalent.

With PES, the ideal of a dialogic science communication is widespread among researchers in the DACH region. This contradicts earlier studies that suggested a dominance of deficit model approaches among science communicators (Ridgway et al., 2020; Simis et al., 2016; Su et al., 2017). Furthermore, consistent with our theoretical assumptions and previous findings (Besley et al., 2018), our results show that scientists’ mental models are consistent with their behaviour: Those who have internalized the Strategic Science Communication model tend to communicate particularly strategically, and also those who have internalized the PUS model or PES model tend to choose forms of communication that fit their mental models. Moreover, many respondents indicate that they have engaged in dialogic science communication (see also Bucchi and Trench, 2014). In the DACH region, this may be a consequence of the programmatic orientation of science policy: funding agencies, for example, have long emphasized the need for dialogic public practices (Schäfer, 2009). Science communication in Germany, Switzerland and Austria is increasingly understood as a process between equals (see also Bucchi and Trench, 2014; Schäfer, 2009; Schmid-Petri and Bürger, 2019).

Our results also show that precarious working conditions such as temporary employment are associated with an internalization of the mental model of Strategic Science Communication. This could indicate a shift in the mental models of academics, especially due to the changing conditions in the academic system, which particularly affect junior academics (e.g. Fang and Casadevall, 2015; Metz-Göckel et al., 2016). Accordingly, science communication may be used as a strategic tool in competition with other academics. Unfortunately, the cross-sectional design of the present study does not allow definitive causal conclusions to be drawn. Future studies could start here and investigate what lies behind the correlations.

As in other areas, gender differences are evident in the mental models of science communication. The mental models of Strategic Science Communication and PES appear to be more internalized in female academics than in male academics. This finding matches Burchell’s (2015) literature review, which states that the level of women’s participation in public engagement may be slightly higher than men’s. The study by Anzivino (2021) also addressed gender differences in public engagement and found for a large national sample of Italian academics that women do local engagement activities but fewer media engagement activities than men. This indicates country differences, which should be investigated more extensively in future cross-country comparative studies.

Older academics tend to adhere quite strongly to the PES model. This supports the observation of Burchell (2015), who emphasizes that academics who are older show more public engagement. In our study of academics in Germany, Austria and Switzerland, we can further confirm what the Pew Research Center (2015) found for American academics: Science communication via social media is more important for younger academics than for older ones.

We also found differences in the respondents’ mental models by country and discipline. Respondents from the humanities, social sciences and life sciences are more likely to adhere to the PES and Strategic Science Communication models than natural scientists, which may explain why participation in public engagement is considerably higher in these disciplines compared with STEM (Burchell, 2015).

Our results also show that there are academics with a strong strategic orientation in their mental models. They are also more strategy-driven in their science communication, rather than driven by social responsibility, the desire to educate or to engage in dialogue. What are the implications of this strategic approach to science communication? On the positive side, potentially more researchers, and especially early-career female researchers, are contributing scientific findings to public discourse. However, this shift also means an additional workload for the often already precariously employed researchers, which is still inappropriately rewarded and, above all, has too little support from HEIs. But is it ethically justifiable to communicate science (only) for personal gain? How does this personal motivation affect the results of communication to the public? Future research could start here.

Our study has limitations: Mental models are cognitive constructs that a person may not always be aware of, so unconscious beliefs about personal science communication may not be fully identified by an online survey. Another limitation of the results is self-selection bias: Respondent participation was voluntary, that is, invited individuals could decide for themselves whether or not to participate in the survey. Individuals motivated to participate may differ systematically in their opinions from the statistical population, which may bias predictions for the population. However, as shown above, all in all our sample seems to be a good representation of the population, which is why we do not suspect any serious biases.

Overall, the dominance of the PES model and the emergence of the strategic science communication mental model among academics in the DACH region seems to indicate a change in the prevailing mental models of science communication. Research shows that HEIs and the academics’ cultural patterns of science communication have traditionally been slow to change (Burchell, 2015). Yeo and Brossard (2017) believe that the shift in cultural attitudes of science towards public communication is driven, on one hand, by funding agencies that require public engagement and outreach as part of grant proposals; in the last decade, they have prioritized such activities through calls for proposals that explicitly explore and implement such projects. On the other hand, the increasing number of scientists interacting with the media and conducting other public engagement activities may in itself be changing the culture of science. Research funders and academics themselves now consistently agree that academics have a duty to communicate with the public (Burchell, 2015). However, it should always be kept in mind that ‘although many researchers are highly committed to public engagement – there is a sense in which the public engagement agenda represents a fundamental, externally-driven redrawing of the academic job description’ (Burchell, 2015: 42).

## Supplemental Material

sj-docx-1-pus-10.1177_09636625211065743 – Supplemental material for Mapping mental models of science communication: How academics in Germany, Austria and Switzerland understand and practice science communication Supplemental material, sj-docx-1-pus-10.1177_09636625211065743 for Mapping mental models of science communication: How academics in Germany, Austria and Switzerland understand and practice science communication by Sabrina Heike Kessler, Mike S. Schäfer, David Johann and Heiko Rauhut in Public Understanding of Science

## Notes

1. Various researchers were involved in the questionnaire with their own research questions. Rauhut et al. (2021b) document the elements of the whole survey and the core item batteries, questions and specifics of the sampling and data-cleaning. Rauhut et al. (2021b) also provide additional material and documents, such as the codebook, ethics approval and data protection.

2. Both employment conditions are significantly correlated with gender (part-time employment:|Cramér’s V*|* = .18, *p* < .001; temporary employment:|Cramér’s V*|* = .14, *p* < .001).

3. SPSS as well as Stata/SE were used for data analysis, including several user-written Stata-commands/packages, such as the *fitstat* command written by J. Scott Long and Jeremy Freese, the *factortest* command written by Joao Pedro Azevedo, as well as the *fre, estadd, estpost, eststo, and esttab* commands written by Ben Jann.

4. For each mental model, we also calculated mean indices of the items used to measure the mental models and tested the mean differences between the mean indices using t-tests. The results reveal that the means of the indices differ significantly (p-value for each pair < .001).

5. To identify how much variance is explained by different bundles of factors, we computed additional models. These are reported in the Supplemental Material (see Tables A1 to A3).
