# Supplementary material for: Mapping mental models of science communication: How academics in Germany, Austria and Switzerland understand and practice science communication
Source: Public Underst Sci. 2022 Jan 11;31(6):711–31. doi: 10.1177/09636625211065743 (PMC9344493; doi:10.1177/09636625211065743)
Supplement: sj-docx-1-pus-10.1177_09636625211065743 – Supplemental material for Mapping mental models of science communication: How academics in Germany, Austria and Switzerland understand and practice science communication [file sj-docx-1-pus-10.1177_09636625211065743.docx]

**Supplemental material**

of the article **«**Mapping mental models of science communication: How academics in Germany, Austria, and Switzerland understand and do science communication **«**

Authors: Sabrina Heike Kessler, Mike S. Schäfer, David Johann, Heiko Rauhut

**Content**

[Table A1: Factors explaining Strategic Science Communication (OLS regression models; unstandardized coefficients) 2](#_Toc87523602)

[Table A2: Factors explaining Public Engagement with Science (OLS regression models; unstandardized coefficients) 4](#_Toc87523603)

[Table A3: Factors explaining Public Understanding of Science (OLS regression models; unstandardized coefficients) 6](#_Toc87523604)

[Table A4: Factors explaining the affiliation with various types of academics revealing different communication behaviours (multinomial logistic regression model; unstandardized coefficients) 8](#_Toc87523605)

[Figure A1: Effect of mental models of science communication on the likelihood of belonging to various types of academics revealing different kinds of communication behaviour (calculation of the predicted probabilities based on the multinomial logistic regression model in Table A4) 10](#_Toc87523606)

# Table A1: Factors explaining Strategic Science Communication (OLS regression models; unstandardized coefficients)

|  | Model 1  (Sociodemographic factors) | Model 2  (Sociodemographic factors + Academic status) | Model 3  (Sociodemographic factors + Academic status +Perceived work situation) | Model 4  (Sociodemographic factors + Academic status +Perceived work situation + Scientific field) |
| --- | --- | --- | --- | --- |
| Female (Ref.: Men) | 0.18*** | 0.16*** | 0.13*** | 0.12*** |
|  | (0.02) | (0.02) | (0.02) | (0.02) |
| 30 to 39 (Ref.: <30) | 0.05 | 0.09* | 0.06 | 0.04 |
|  | (0.03) | (0.04) | (0.04) | (0.04) |
| 40 to 49 (Ref.: <30) | -0.03 | 0.09* | 0.05 | 0.02 |
|  | (0.04) | (0.05) | (0.05) | (0.05) |
| 50 to 59 (Ref.: <30) | -0.11** | 0.08 | 0.01 | -0.02 |
|  | (0.04) | (0.05) | (0.05) | (0.05) |
| 60+ (Ref.: <30) | -0.06 | 0.13* | 0.05 | 0.02 |
|  | (0.05) | (0.06) | (0.06) | (0.06) |
| Austria (Ref.: Germany) | 0.20*** | 0.21*** | 0.19*** | 0.19*** |
|  | (0.03) | (0.03) | (0.03) | (0.03) |
| Switzerland (Ref.: Germany) | 0.26*** | 0.27*** | 0.25*** | 0.25*** |
|  | (0.03) | (0.03) | (0.03) | (0.03) |
| Postdoc (Ref.: Predoc) |  | -0.05 | -0.14*** | -0.11*** |
|  |  | (0.03) | (0.03) | (0.03) |
| Professor (Ref.: Predoc) |  | -0.06 | -0.16*** | -0.13** |
|  |  | (0.05) | (0.04) | (0.05) |
| Tenured (Ref.: Not tenured) |  | -0.16*** | -0.12*** | -0.11** |
|  |  | (0.04) | (0.03) | (0.03) |
| Part-time (Ref.: Full-time) |  | 0.02 | 0.02 | 0.02 |
|  |  | (0.03) | (0.03) | (0.03) |
| Discrepancy between desired time for research and |  |  | -0.39*** | -0.41*** |
| time actually available for research |  |  | (0.05) | (0.05) |
| Autonomy |  |  | -0.04*** | -0.04*** |
|  |  |  | (0.01) | (0.01) |
| Sense foundation |  |  | 0.12*** | 0.12*** |
|  |  |  | (0.01) | (0.01) |
| Competition |  |  | 0.05*** | 0.06*** |
|  |  |  | (0.01) | (0.01) |
| Workload |  |  | 0.01 | 0.01 |
|  |  |  | (0.01) | (0.01) |
| Pressure to publish |  |  | -0.03** | -0.03* |
|  |  |  | (0.01) | (0.01) |
| Pressure to win grants |  |  | 0.08*** | 0.08*** |
|  |  |  | (0.01) | (0.01) |
| Humanities (Ref.: Natural sciences) |  |  |  | 0.21*** |
|  |  |  |  | (0.04) |
| Social sciences (Ref.: Natural sciences) |  |  |  | 0.14*** |
|  |  |  |  | (0.04) |
| Life sciences (Ref.: Natural sciences) |  |  |  | 0.13*** |
|  |  |  |  | (0.04) |
| Engineering (Ref.: Natural sciences) |  |  |  | 0.24*** |
|  |  |  |  | (0.04) |
| Other (Ref.: Natural sciences) |  |  |  | 0.31*** |
|  |  |  |  | (0.05) |
| Constant | -0.19*** | -0.19*** | -0.85*** | -1.01*** |
|  | (0.03) | (0.03) | (0.08) | (0.08) |
| *N* | 7592 | 7592 | 7592 | 7592 |
| Adj. *R*2 | 0.02 | 0.03 | 0.07 | 0.08 |

Notes: Standard errors in parentheses. * *p* < .05, ** *p* < .01, *** *p* < .001

# Table A2: Factors explaining Public Engagement with Science (OLS regression models; unstandardized coefficients)

|  | Model 1  (Sociodemographic factors) | Model 2  (Sociodemographic factors + Academic status) | Model 3  (Sociodemographic factors + Academic status +Perceived work situation) | Model 4  (Sociodemographic factors + Academic status +Perceived work situation + Scientific field) |
| --- | --- | --- | --- | --- |
| Female (Ref.: Men) | 0.22*** | 0.20*** | 0.20*** | 0.14*** |
|  | (0.02) | (0.02) | (0.02) | (0.02) |
| 30 to 39 (Ref.: <30) | 0.04 | 0.10** | 0.12** | 0.07 |
|  | (0.03) | (0.04) | (0.04) | (0.04) |
| 40 to 49 (Ref.: <30) | 0.15*** | 0.24*** | 0.27*** | 0.19*** |
|  | (0.04) | (0.05) | (0.05) | (0.05) |
| 50 to 59 (Ref.: <30) | 0.29*** | 0.39*** | 0.40*** | 0.33*** |
|  | (0.04) | (0.05) | (0.05) | (0.05) |
| 60+ (Ref.: <30) | 0.36*** | 0.46*** | 0.46*** | 0.39*** |
|  | (0.05) | (0.06) | (0.06) | (0.06) |
| Austria (Ref.: Germany) | 0.02 | 0.02 | 0.00 | -0.00 |
|  | (0.03) | (0.03) | (0.03) | (0.03) |
| Switzerland (Ref.: Germany) | 0.08** | 0.08** | 0.05 | 0.04 |
|  | (0.03) | (0.03) | (0.03) | (0.03) |
| Postdoc (Ref.: Predoc) |  | -0.08* | -0.05 | -0.05 |
|  |  | (0.03) | (0.03) | (0.03) |
| Professor (Ref.: Predoc) |  | -0.07 | -0.04 | -0.07 |
|  |  | (0.05) | (0.05) | (0.05) |
| Tenured (Ref.: Not tenured) |  | 0.00 | 0.01 | 0.03 |
|  |  | (0.04) | (0.04) | (0.04) |
| Part-time (Ref.: Full-time) |  | 0.11*** | 0.11*** | 0.04 |
|  |  | (0.03) | (0.03) | (0.03) |
| Discrepancy between desired time for research and |  |  | -0.40*** | -0.44*** |
| time actually available for research |  |  | (0.05) | (0.05) |
| Autonomy |  |  | 0.01 | -0.00 |
|  |  |  | (0.01) | (0.01) |
| Sense foundation |  |  | 0.03*** | 0.03** |
|  |  |  | (0.01) | (0.01) |
| Competition |  |  | -0.01 | -0.02* |
|  |  |  | (0.01) | (0.01) |
| Workload |  |  | 0.02** | 0.02** |
|  |  |  | (0.01) | (0.01) |
| Pressure to publish |  |  | -0.01 | -0.01 |
|  |  |  | (0.01) | (0.01) |
| Pressure to win grants |  |  | -0.03** | -0.02 |
|  |  |  | (0.01) | (0.01) |
| Humanities (Ref.: Natural sciences) |  |  |  | 0.32*** |
|  |  |  |  | (0.04) |
| Social sciences (Ref.: Natural sciences) |  |  |  | 0.37*** |
|  |  |  |  | (0.04) |
| Life sciences (Ref.: Natural sciences) |  |  |  | 0.09* |
|  |  |  |  | (0.04) |
| Engineering (Ref.: Natural sciences) |  |  |  | -0.01 |
|  |  |  |  | (0.04) |
| Other (Ref.: Natural sciences) |  |  |  | 0.30*** |
|  |  |  |  | (0.05) |
| Constant | -0.23*** | -0.28*** | -0.31*** | -0.36*** |
|  | (0.03) | (0.03) | (0.08) | (0.08) |
| *N* | 7592 | 7592 | 7592 | 7592 |
| Adj. *R*2 | 0.02 | 0.03 | 0.04 | 0.06 |

Notes: Standard errors in parentheses. * *p* < .05, ** *p* < .01, *** *p* < .001

# Table A3: Factors explaining Public Understanding of Science (OLS regression models; unstandardized coefficients)

|  | Model 1  (Sociodemographic factors) | Model 2  (Sociodemographic factors + Academic status) | Model 3  (Sociodemographic factors + Academic status +Perceived work situation) | Model 4  (Sociodemographic factors + Academic status +Perceived work situation + Scientific field) |
| --- | --- | --- | --- | --- |
| Female (Ref.: Men) | 0.01 | 0.01 | -0.01 | -0.00 |
|  | (0.02) | (0.02) | (0.02) | (0.02) |
| 30 to 39 (Ref.: <30) | 0.00 | -0.00 | -0.02 | -0.00 |
|  | (0.03) | (0.04) | (0.04) | (0.04) |
| 40 to 49 (Ref.: <30) | 0.02 | -0.02 | -0.04 | -0.02 |
|  | (0.04) | (0.05) | (0.05) | (0.05) |
| 50 to 59 (Ref.: <30) | 0.10* | 0.02 | -0.02 | 0.00 |
|  | (0.04) | (0.05) | (0.05) | (0.05) |
| 60+ (Ref.: <30) | 0.11* | 0.03 | -0.02 | -0.00 |
|  | (0.05) | (0.06) | (0.06) | (0.06) |
| Austria (Ref.: Germany) | 0.08* | 0.07* | 0.05 | 0.04 |
|  | (0.03) | (0.03) | (0.03) | (0.03) |
| Switzerland (Ref.: Germany) | 0.04 | 0.03 | 0.02 | 0.01 |
|  | (0.03) | (0.03) | (0.03) | (0.03) |
| Postdoc (Ref.: Predoc) |  | 0.00 | -0.05 | -0.06 |
|  |  | (0.03) | (0.03) | (0.03) |
| Professor (Ref.: Predoc) |  | 0.04 | -0.05 | -0.04 |
|  |  | (0.05) | (0.05) | (0.05) |
| Tenured (Ref.: Not tenured) |  | 0.06 | 0.07* | 0.06 |
|  |  | (0.04) | (0.04) | (0.04) |
| Part-time (Ref.: Full-time) |  | 0.01 | 0.03 | 0.05 |
|  |  | (0.03) | (0.03) | (0.03) |
| Discrepancy between desired time for research and |  |  | -0.19*** | -0.15** |
| time actually available for research |  |  | (0.05) | (0.05) |
| Autonomy |  |  | 0.01 | 0.02 |
|  |  |  | (0.01) | (0.01) |
| Sense foundation |  |  | 0.14*** | 0.14*** |
|  |  |  | (0.01) | (0.01) |
| Competition |  |  | -0.00 | 0.00 |
|  |  |  | (0.01) | (0.01) |
| Workload |  |  | 0.03*** | 0.03*** |
|  |  |  | (0.01) | (0.01) |
| Pressure to publish |  |  | 0.03** | 0.03* |
|  |  |  | (0.01) | (0.01) |
| Pressure to win grants |  |  | 0.01 | 0.00 |
|  |  |  | (0.01) | (0.01) |
| Humanities (Ref.: Natural sciences) |  |  |  | -0.13*** |
|  |  |  |  | (0.04) |
| Social sciences (Ref.: Natural sciences) |  |  |  | -0.06 |
|  |  |  |  | (0.04) |
| Life sciences (Ref.: Natural sciences) |  |  |  | 0.12*** |
|  |  |  |  | (0.04) |
| Engineering (Ref.: Natural sciences) |  |  |  | -0.01 |
|  |  |  |  | (0.04) |
| Other (Ref.: Natural sciences) |  |  |  | 0.05 |
|  |  |  |  | (0.05) |
| Constant | -0.06 | -0.06 | -1.02*** | -1.03*** |
|  | (0.03) | (0.03) | (0.08) | (0.09) |
| *N* | 7592 | 7592 | 7592 | 7592 |
| Adj. *R*2 | 0.00 | 0.00 | 0.04 | 0.04 |

Notes: Standard errors in parentheses. * *p* < .05, ** *p* < .01, *** *p* < .001

# Table A4: Factors explaining the affiliation with various types of academics revealing different communication behaviours (multinomial logistic regression model; unstandardized coefficients)

|  | (Base outcome: Non-strategic) | |
| --- | --- | --- |
|  | Exclusively strategic | Strategic + PES and/or PUS |
| Female (Ref.: Men) | -0.20* | -0.26*** |
|  | (0.10) | (0.06) |
| 30 to 39 (Ref.: <30) | -0.13 | -0.08 |
|  | (0.14) | (0.09) |
| 40 to 49 (Ref.: <30) | -0.42* | -0.31* |
|  | (0.19) | (0.12) |
| 50 to 59 (Ref.: <30) | -1.24*** | -0.83*** |
|  | (0.23) | (0.14) |
| 60+ (Ref.: <30) | -1.70*** | -1.23*** |
|  | (0.31) | (0.17) |
| Austria (Ref.: Germany) | 0.16 | 0.22** |
|  | (0.13) | (0.08) |
| Switzerland (Ref.: Germany) | 0.29** | 0.21** |
|  | (0.11) | (0.07) |
| Postdoc (Ref.: Predoc) | 0.42** | 0.27** |
|  | (0.13) | (0.08) |
| Professor (Ref.: Predoc) | 0.77*** | 0.39*** |
|  | (0.19) | (0.12) |
| Tenured (Ref.: Not tenured) | -0.11 | -0.06 |
|  | (0.14) | (0.09) |
| Part-time (Ref.: Full-time) | -0.14 | 0.05 |
|  | (0.11) | (0.07) |
| Discrepancy between desired time for research and | -0.13 | 0.08 |
| time actually available for research | (0.21) | (0.14) |
| Autonomy | -0.11** | -0.06* |
|  | (0.04) | (0.03) |
| Sense foundation | 0.01 | 0.07** |
|  | (0.04) | (0.03) |
| Competition | 0.19*** | 0.08*** |
|  | (0.04) | (0.02) |
| Workload | 0.00 | 0.06** |
|  | (0.04) | (0.02) |
| Pressure to publish | -0.07 | -0.05 |
|  | (0.05) | (0.03) |
| Pressure to win grants | -0.05 | 0.03 |
|  | (0.04) | (0.02) |
| Humanities (Ref.: Natural sciences) | 0.24 | 0.32** |
|  | (0.17) | (0.10) |
| Social sciences (Ref.: Natural sciences) | 0.45** | 0.38*** |
|  | (0.15) | (0.09) |
| Life sciences (Ref.: Natural sciences) | 0.32* | 0.02 |
|  | (0.16) | (0.10) |
| Engineering (Ref.: Natural sciences) | 0.49** | 0.09 |
|  | (0.17) | (0.11) |
| Other (Ref.: Natural sciences) | 0.29 | 0.40** |
|  | (0.21) | (0.12) |
| Mental model Strategic Science | 0.22*** | 0.59*** |
| Communication (factor scores) | (0.05) | (0.03) |
| Mental model PES (factor scores) | -0.06 | 0.28*** |
|  | (0.05) | (0.03) |
| Mental model PUS (factor scores) | -0.21*** | 0.26*** |
|  | (0.05) | (0.03) |
| Constant | -1.95*** | -1.43*** |
|  | (0.35) | (0.23) |
| *N* | 6947 |  |
| Cragg-Uhler R2 | 0.16 |  |
| McFadden's Adj R2 | 0.09 |  |

Notes: Standard errors in parentheses. * p<.05, ** p<.01, *** p<.001

# Figure A1: Effect of mental models of science communication on the likelihood of belonging to various types of academics revealing different kinds of communication behaviour (calculation of the predicted probabilities based on the multinomial logistic regression model in Table A4)


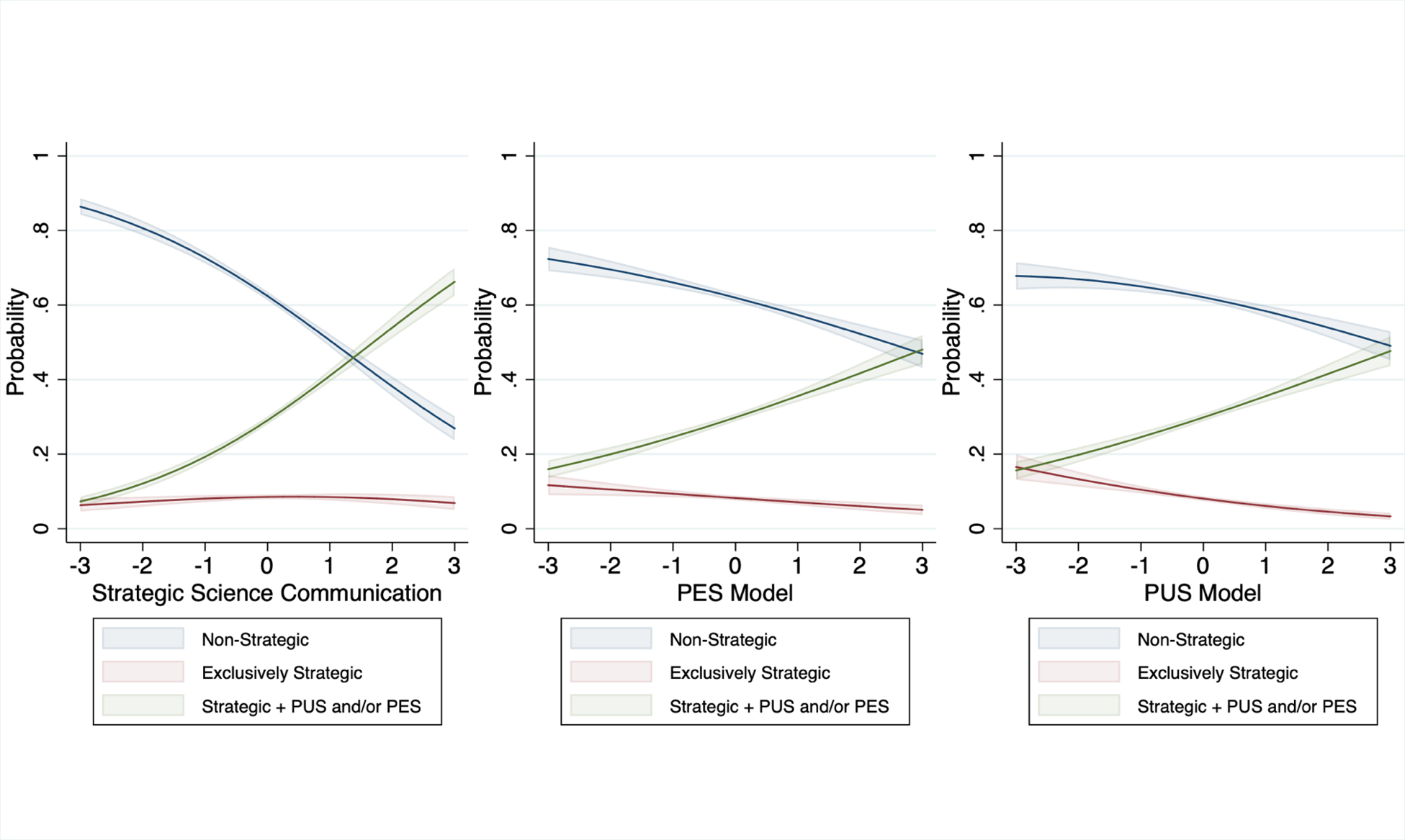


Notes: We used the “observed-value approach” to calculate the predicted probabilities (Hanmer and Ozan Kalkan, 2013).
